# Novel Frameshift Heterozygous Mutation in *UBAP1* Gene Causing Spastic Paraplegia-80: Case Report With Literature Review

**DOI:** 10.3389/fneur.2022.820202

**Published:** 2022-03-07

**Authors:** Chao Zhang, Xiaowei Zhu, Zeyu Zhu, Ruilong Ni, Taotao Liu, Haoran Zheng, Shihua Liu, Li Cao, Ping Zhong, Wotu Tian

**Affiliations:** ^1^Suzhou Hospital of Anhui Medical University Suzhou Municipal Hospital of Anhui Province, Suzou, China; ^2^Department of Neurology, Shanghai Jiao Tong University Affiliated Sixth People's Hospital, Shanghai, China; ^3^School of Medicine, Anhui University of Science and Technology, Huainan, China

**Keywords:** hereditary spastic paraplegia, spastic paraplegia-80, *UBAP1*, whole exome sequencing, case report

## Abstract

Hereditary spastic paraplegia (HSP) represents a group of rare inherited neurodegenerative conditions and is characterized by progressive lower limb spasticity. Ubiquitin-associated protein 1 (*UBAP1*)-related HSP is classified as spastic paraplegia-80 (SPG80), which is an autosomal-dominant (AD) juvenile-onset neurologic disorder and mainly affects the lower limbs. We described the clinical and genetic features of two patients in the same family caused by heterozygous mutation of the *UBAP1* gene. The proband was a 34-year-old woman with progressive spasticity and hyperreflexia in the lower limbs for 26 years. Her mother also had similar symptoms since the age of 6. The proband and her mother only had motor dysfunctions, such as unsteady gait, hypertonia, and hyperreflexia of lower limbs. Other system functions (sensory, urinary, visual, and cognitive impairments) were not involved. WES disclosed a frameshift mutation (c.371dupT) in the *UBAP1* gene, which was predicted to be “likely pathogenic” and was co-segregated in the pedigree. c.371dupT, encoding the truncated UBAP1 protein with 72.6% missing of the normal amino acid sequence, is responsible for the spastic paraplegia (SPG) in this family. In combination with clinical characteristics, genetic testing results, and co-segregation analysis, the diagnosis is considered to be pure spastic paraplegia-80 (SPG80), which is an AD disease. By retrospectively analyzing the documented cases, we comprehensively review the phenotypic features and summarize the genotype spectrum of SPG80 to enhance earlier recognition and therapeutic strategies.

## Introduction

Hereditary spastic paraplegia (HSP) represents a group of rare inherited neurodegenerative conditions and is characterized by progressive lower limb spasticity (1). HSP is traditionally classified into complicated and uncomplicated forms according to the presence of additional clinical features or not, such as cognitive decline, cerebellar ataxia, peripheral neuropathy, or parkinsonism. The estimated prevalence of HSP is about 1.2–9.6/100,000 in the general population (2, 3). To date, more than 82 genes have been associated with HSP under various modes of inheritance, such as autosomal recessive (AR), autosomal dominant (AD), X-linked, and maternally inherited (mitochondrial) (2, 4, 5). In 2019, Farazi Fard et al. first described the association between ubiquitin-associated protein 1 gene (*UBAP1*) and HSP (6). *UBAP1*-related-HSP is classified as spastic paraplegia-80 (SPG80), which is an AD juvenile-onset neurologic disorder and mainly affects the lower limbs. Among all the cases published before, the most common phenotype is the pure form, which is characterized by progressive spasticity and hyperreflexia, but other systems are not involved (7, 8). However, some patients indeed manifest as complicated form, neurological or other features are also present, such as cerebellar ataxia and mild cognitive impairment (6, 9).

Here, we reported two patients from a Chinese family with SPG80 due to a novel heterozygous frameshift mutation in the *UBAP1* gene. On the basis of thorough clinical and genetic analysis, we perform a comprehensive review and summarization of previously reported SPG80 cases to strengthen the understanding and clinical diagnosis of this disease.

## Case Description

**Figure 1:**
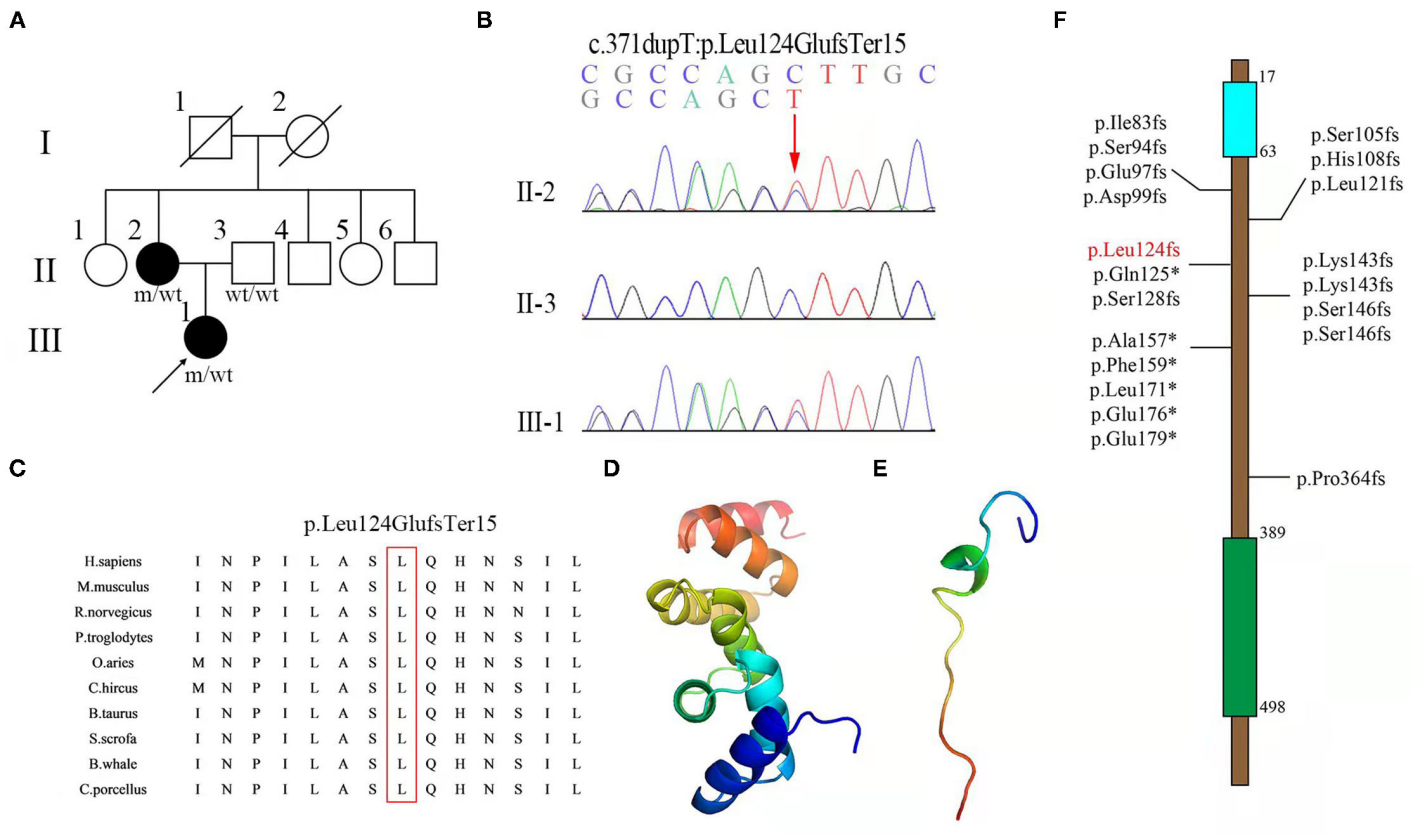
**(A)** Pedigree of a family with hereditary spastic paraplegia (HSP), with the indicated frameshift mutation of *UBAP1*. Squares and circles indicate men and women, respectively. Black symbols represent members with an HSP phenotype, and empty symbols represent unaffected individuals. The arrows indicate patients; wt indicate wide-type; m indicates mutation. **(B)** Sequence chromatogram showing the c.371dupT variant in UBAP1 in the family. **(C)** Conservatism analysis of the 124th amino acid among different species. Structures of **(D)** wild-type and **(E)** mutant (Leu124GlufsTer15) of the *UBAP1* protein were predicted with the Phyre2 web portal. **(F)** Documented mutations identified in spastic paraplegia-80 (SPG80). The numbers indicate the locations of mutations in protein. Blue and green rectangles indicate the UMA and SOUBA domains, respectively. The mutation found in this study is marked in red.

We enrolled a Han Chinese family (Figure 1A) without consanguineous history in this study, with 2 patients fulfilling the clinical diagnosis of pure HSP and 5 unaffected individuals (10). The proband and her family members were clinically examined. The proband (III1 in Figure 1A) presented with childhood-onset abnormal gait. She was 34 years old with progressive difficulty in walking for 26 years. The disease progressed gradually. At the age of 8, the muscles of lower limbs became stiff, tight, and swollen when walking. Subsequently, the lower limb weakness led to laborious leg-lifting. During junior high school days, she became easy to fall and could not walk independently. Then, frequent stumbling and gait disorders were recorded. At the age of 34, the physical examination showed normal strength in neck flexion and upper limbs (5/5 on a medical research council scale graded 0–5) but reduced strength of lower limb abduction (4/5). Hypertonia, hyperreflexia, and ankle clonus in the lower limbs were also disclosed. Muscle atrophies in the four limbs were not noticed. She walked slowly with a scissor gait. Saccadic pursuit, nystagmus, myalgia, muscular atrophy, dysphagia, ataxia, and cognitive impairment were not noted. Brain magnetic resonance imaging (MRI) performed at age 14 was normal.

The other affected within the same family was the 60-year-old mother (II2 in Figure 1A) of the proband described above who had manifestations of similar clinical features. She started to suffer from progressive walking difficulty at the age of 6, but she could walk without aid until the age of 12. The spastic gait continued to progress moderately. At the time of recent visit, she could still walk with aid. Physical examination showed normal strength in neck flexion and upper limbs (5/5), reduced strength in lower limb abduction (3/5), hypertonia, hyperreflexia, and ankle clonus in the lower limbs.

After admission, relevant examinations were carried out. No abnormality was revealed by blood routine, routine urine and stool testing, biochemical indicators, coagulation function, thyroid function, glycosylated hemoglobin, inflammatory markers, and tumor markers in the two patients.

Whole exome sequencing was performed using DNA from the proband's peripheral blood sample, thus showing the heterozygous variant c.371dupT (p.Leu124GlufsTer15) in the *UBAP1* gene. Sanger sequencing and co-segregation were further performed on the family, including the proband, the affected mother (c.371dupT), and the father (normal) (Figure 1B). The site of mutation was not identified in 1,000 Genomes (http://browser.1000genomes.org), Genome Aggregation Database dataset 2.1.1 (https://gnomad.broadinstitute.org), and the Exome Aggregation Consortium dataset (http://exac.broadinstitute.org) as well as in 200 healthy controls. This variant was not documented in the Human Gene Mutation Database (HGMD) and was predicted to be “disease-causing” by MutationTaster (http://www.mutationtaster.org, probability score: 1, range: 0–1). The site of mutation was well-conserved among different species (Figure 1C). Structures of wild-type and mutant UBAP1 proteins were predicted with the Phyre2 web portal (http://www.sbg.bio.ic.ac.uk/phyre2) (Figures 1D,E). c.371dupT encoded the truncated UBAP1 protein with 72.6% missing of the normal amino acid sequence. Thus, the variant was classified as “likely pathogenic” according to the American College of Medical Genetics and Genomics (ACMG) standards and guidelines (PVS1, PM2) (11). In combination with the evidence of clinical characteristics and genetic testing results, it was diagnosed as pure SPG80. SPG80 needs to be differentiated from spinocerebellar ataxia, peripheral neuropathy, parkinsonism, and other subtypes of hereditary spastic paraplegias.

## Discussion

**Figure 2:**
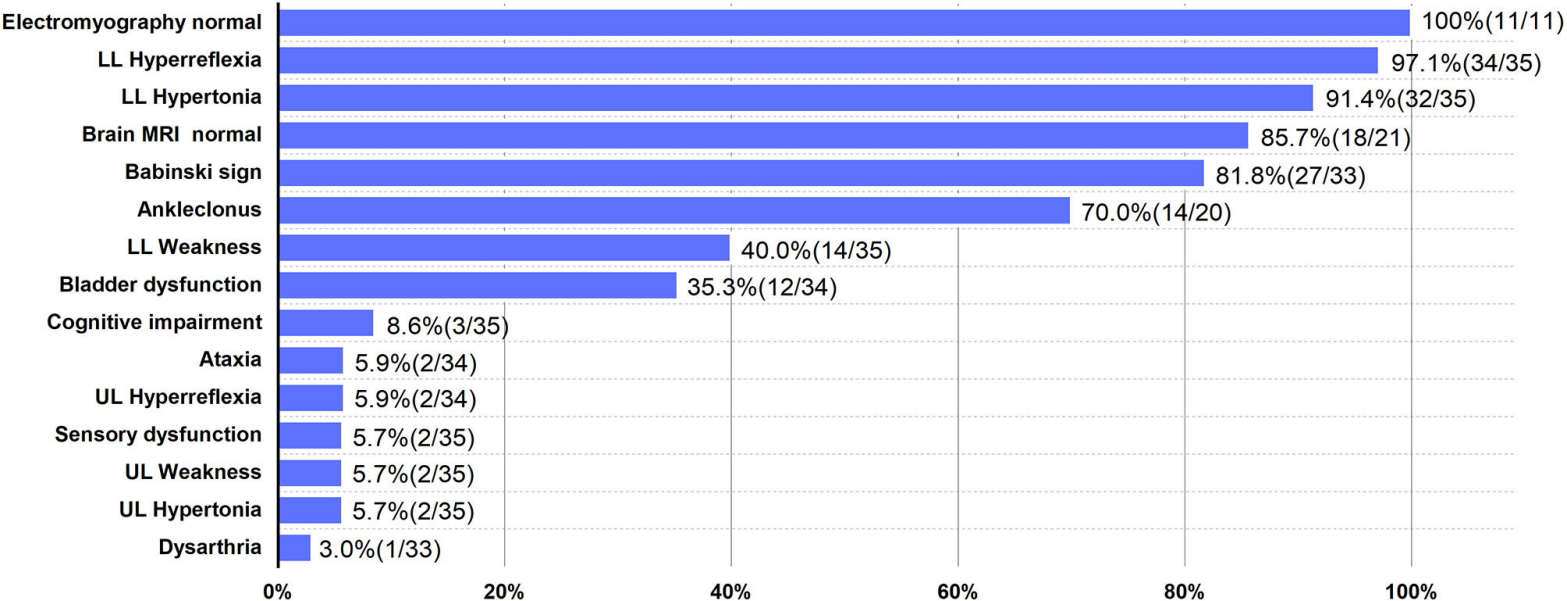
Clinical features of SPG80 probands with *UBAP1* mutations. For each clinical manifestation, the proportion of patients is indicated. UL, upper limbs; LL, lower limbs.

We demonstrated a novel mutation in the *UBAP1* gene in a family with clinical diagnosis of SPG80. *UBAP1* (NM_016525. 5), located on chromosome 9p13, is composed of 16 exons spanning 73.5 kb. To date, a total of twenty heterozygous *UBAP1* mutations have been identified among 91 patients from 35 families worldwide and diagnosed as SPG80 (including this one). Nineteen mutations (c.247_248insGTGAATTC, c.279delG, c.286_290dupCCAGA, c.295dupG, c.312delC, c.324_325delCA, c.361dupC, c.373C>T, c.382delA, c.425_426delAG, c.426_427delGA, c.436_437insTGAG, c.468_469delTG, c.437dupG, c.475_476delTT, c.512T>G, c.526G>T, c.535G>T, and c.1091delC) have been reported (6–9, 12–14), while our mutation (c.371dupT) was undocumented before (Figure 1F). Among the 20 mutations, 16 were frameshift ones identified in 67 patients, of which 53 showed a pure type, 12 showed a complicated type, and 2 were asymptomatic cases. Another 4 were non-sense mutations detected in 24 cases, of which 23 were pure form and 1 was asymptomatic. The most frequent mutation identified so far by all studies was c.425_426delAG, reported in 11 families from different sources, and could lead to 3 different phenotypes. Among all the 91 patients, the number of patients with pure (onset age = 11 years old) and complicated (onset age = 6 years old) forms was 76 and 12, respectively. Female to male ratio was 2.14 (62:29), suggesting a female predilection of this disorder. Among all the families, the probands displayed classic symptoms of progressive lower-limb spastic paraplegia, including hypertonia (32/35), hyperreflexia (34/35), ankle clonus (14/20), and Babinski sign (27/33). However, the relatively uncommon phenotypes were also presented in several cases, such as hypertonia (2/35) and hyperreflexia (2/34) in the upper limbs, cognitive impairment (3/35), ataxia (2/34), sensory dysfunction (2/35), and dysarthria (1/33). According to results reported before, brain MRI was normal in most patients except for one who presented with mild generalized cortical-subcortical volume loss. Moreover, electromyography results of all the patients were normal (6–9, 12–14). The clinical features of SPG80 probands with *UBAP1* mutations are summarized in Figure 2. Although there are still no clear genotype-phenotype correlations for SPG80 (12), we proposed that clinical heterogeneity may be related to various effects of different *UBAP1* gene mutations on molecular biological function.

The UBAP1 gene encodes the UBAP1 protein, a 502-amino-acid-residue peptide (14). The UBAP1 protein is a subunit of the endosomal sorting complex required for transport-I (ESCRT-I), which comprises TSG101, VPS28, VPS37A, and MVB12 (15, 16). The *UBAP1* protein has a total of 502 amino acids, including 3 main domains, namely, the UMA (UBAP1-MVB12-associated) domain in the N-terminal region (17–63 aa) and the SOUBA (solenoid of overlapping ubiquitin-associated domains) domain in the C-terminal region (389–498 aa) (6). All these variants, including ours, are located between domain UMA and domain SOUBA, indicating that the area is a hotspot region (Figure 1F). A total of 16 *UBAP1* mutations with frameshift were predicted to generate prematurely truncated proteins with complete loss of the SOUBA domain in the C-terminal region of *UBAP1* (7). Previous genetic knockdown experiments on zebrafish have indicated that UBAP1 could disturb its functions in endosomal trafficking, cause abnormal organismal morphology, inhibit motor-neuron outgrowth, and decrease mobility (13). Functional studies on mouse hippocampus revealed that C-terminal deletion of the UBAP1 protein may perturb endosomal fusion and ubiquitinated cargo sorting in relative neurons (8). UBAP1 plays an important role in proteasomal degradation of ubiquitinated cell-surface proteins (15–18). These studies provided genetic and biochemical evidence that mutations in *UBAP1* exert negative effects on protein degradation in the neurological system.

However, the concrete mechanism and detailed function of *UBAP1* deserve further investigations to gain an in-depth understanding of the pathogenesis of SPG80. Furthermore, to clearly define the phenotype–genotype correlations, more patients need to be studied to clarify whether specific mutations are prone to certain clinical characteristics.

## Conclusion

In summary, we reported a new family with SPG80 due to a novel frameshift mutation, c.371dupT, of *UBAP1*. This study further expanded our knowledge of the phenotype and genotype of SPG80.

## Data Availability Statement

The datasets presented in this article are not readily available due to ethical and privacy restrictions. Requests to access the datasets should be directed to the corresponding author.

## Ethics Statement

The studies involving human participants were reviewed and approved by the Ethics Committee of Shanghai Jiao Tong University Affiliated Sixth People's Hospital. The patients/participants provided their written informed consent to participate in this study. Written informed consent was obtained from the individual(s) for the publication of any potentially identifiable images or data included in this article.

## Author Contributions

CZ and XZ contributed to data collection and drafted the manuscript. ZZ, TL, HZ, and SL contributed to analysis and interpretation of data and statistical analysis. LC contributed to funding, study design and conceptualization, and manuscript revision. PZ and WT contributed to data collection and evaluation, supervision, manuscript revision, and final approval. All authors contributed to the article and approved the submitted version.

## Funding

This work was supported by the National Natural Science Foundation of China (No. 81870889 and No. 81571086), National Key R&D Program of China (No. 2017YFC1310200), Shanghai Municipal Education Commission-Gao Feng Clinical Medicine Grant (No. 20161401), Interdisciplinary Project of Shanghai Jiao Tong University (No. YG2016MS64), and Natural Science Foundation of Anhui Medical University (No. 2020xkj088).

## Conflict of Interest

LC is in charge of National Natural Science Foundation of China (No. 81870889 and No. 81571086), National Key R&D Program of China (No. 2017YFC1310200), Shanghai Municipal Education Commission-Gao Feng Clinical Medicine Grant (No. 20161401), and Interdisciplinary Project of Shanghai Jiao Tong University (No. YG2016MS64). CZ is in charge of the Natural Science Foundation of Anhui Medical University (No. 2020xkj088). The remaining authors declare that the research was conducted in the absence of any commercial or financial relationships that could be construed as a potential conflict of interest.

## Publisher's Note

All claims expressed in this article are solely those of the authors and do not necessarily represent those of their affiliated organizations, or those of the publisher, the editors and the reviewers. Any product that may be evaluated in this article, or claim that may be made by its manufacturer, is not guaranteed or endorsed by the publisher.
